# Multiarm Star-Shaped Polydimethylsiloxanes with a Dendritic Branching Center

**DOI:** 10.3390/molecules26113280

**Published:** 2021-05-29

**Authors:** Pavel A. Tikhonov, Nataliya G. Vasilenko, Marat O. Gallyamov, Georgii V. Cherkaev, Viktor G. Vasil’ev, Nina V. Demchenko, Mikhail I. Buzin, Sergey G. Vasil’ev, Aziz M. Muzafarov

**Affiliations:** 1 Enikolopov Institute of Synthetic Polymeric Materials, Russian Academy of Sciences, 117393 Moscow, Russia; tikhonpa@ispm.ru (P.A.T.); georgij.cherkaev@gmail.com (G.V.C.); nika1946@mail.ru (N.V.D.); aziz@ispm.ru (A.M.M.); 2 Nesmeyanov Institute of Organoelement Compounds, Russian Academy of Sciences, 119991 Moscow, Russia; glm@polly.phys.msu.ru (M.O.G.); viktor@ineos.ac.ru (V.G.V.); buzin@ineos.ac.ru (M.I.B.); 3 Faculty of Physics, Lomonosov Moscow State University, 119991 Moscow, Russia; 4 Institute of Problems of Chemical Physics, Russian Academy of Sciences, Academician Semeonov av. 1, Chernogolovka, 142432 Moscow, Russia; viesssw@mail.ru

**Keywords:** polylithium initiator, multiarm stars, anionic polymerization of hexamethylcyclotrisiloxane, carbosilane dendrimers

## Abstract

New multiarm stars have been synthesized based on polylithium derivatives of high-generation carbosilane dendrimers. In the synthesis of multiarm stars based on the eighth-generation dendrimer, steric hindrances were observed even during the synthesis of a polylithium initiator. Subsequently, this led to chain transfer reactions between growing arms, as well as other side effects. As a result, dense nanogel formations with a higher tendency of ordering than in classical objects of this type were isolated from the reaction mixture. The study of the rheology of multiarm stars based on sixth-generation dendrimers made it possible to determine the activation energies of viscous flow in these objects, which makes it possible to consider them as objects with a macromolecular nature and a reptation flow mechanism.

## 1. Introduction

The anionic polymerization of organocyclosiloxanes, especially in the nonequilibrium variant, makes it possible not only to obtain silicone rubbers, whose production is currently estimated in the hundreds of thousands of tons, but also different macromolecular systems of various designs [1,2,3,4,5,6,7,8,9,10]. We dedicate this work to Chojnowski, whose large contribution to this area, including in terms of the synthesis of various multiarm systems, cannot be overestimated [11,12,13].

Star-shaped polydimethylsiloxane polymers have a long history; the first works on obtaining three- and four-armed systems were carried out in the 1960s in Kraus’ [14] and Andrianov’s [7] laboratories. At that time, researchers regarded them primarily as tools for the formation of defect-free polymer networks, but this approach was postponed for a long time due to the imperfection of the synthetic methods for such structures. The modern approach to the synthesis and research of star-shaped polydimethylsiloxanes was much more widespread and prepared. In this case, the real transition to precise multiarm stars occurred because of the appearance of dendrimers, with their practically unlimited and, at the same time, definite number of functional groups [15,16]. The work on synthesizing such structures was carried out by Roovers [17], Muzafarov [4], and Gnanou [18] and was largely initiated by the theoretical studies by Daoud and Cotton [19]. This time, the researchers were interested in the unusual rheology of stars, i.e., a decrease of their solution and melt viscosities upon an increase in the number of arms. Roovers and Hadjichristidis synthesized the first star-shaped structures using carbosilane dendrimers as branching centers, and they found that the high functionality of the initial branching center is a necessary but insufficient condition as the problem of the complete substitution of the center active groups by oligomeric arms in the case of their large number is not easy. Various strategies for multiarm star synthesis have been developed: convergent (arm-first) and divergent (core-first), as well as a combination of these two approaches [20]. The structures obtained with their help had properties so different from classical polymers that it was proposed to refer to them as macromolecular nanoobjects, or macromolecular particles, along with dendrimers, dense molecular brushes, and nanogels [21,22]. The boundary between low-arm stars that belong to branched polymers and multiarm stars can be determined using the α coefficient of the Mark–Kuhn–Houwink equation [η] = K_η_M^α^. Its magnitude and, more importantly, the absence of the dependence of its magnitude on the molecular weight of the arm within the experimentally obtained values make it possible to unambiguously refer to the new objects as one or another classification group [23,24,25,26]. If a star-shaped polymer has a number of arms f > 30, the value of the α coefficient decreases with the number of arms to very low values for such systems (at f > 100, α = 0.06) [24], which characterizes the object as a rigid and compact globule. The nature of these specific polymeric macromolecules determines the peculiarities of their behavior. A number of papers [27,28] have distinguish two limiting cases in star-shaped systems: the first type is stars with a small number of long arms and a low-volume core that remains permeable to elements of the neighboring stars and do not become arranged, and their terminal dynamics are mainly controlled by the relaxation of the arms. The second type is stars with a large number of short arms and a core that is impermeable to the elements of the neighboring stars, which show a clear arrangement and where the slowest dynamics are controlled by structural rearrangements. The first group can be regarded as a typical polymer system, but the second one demonstrates analogies with colloids. The studies were carried out on examples of polyisoprene and polybutadiene stars.

This work is an extension of the studies on star-shaped polydimethylsiloxanes (PDMSs) with a different number of arms [5,29,30,31]. It was previously shown that PDMS stars with a number of arms f > 30, based on an examination of their diluted solutions, refer to dense globular objects with α = 0.06–0.15. A study on PDMS star rheology showed the Newtonian nature of the flow in systems with f = 8 and 32, and a pseudoplastic flow at f = 128 [31]. In this case, the viscous flow activation energy value E_act_ of all the synthesized samples, regardless of the number of arms, insignificantly differed from the E_act_ of linear PDMS [32], which indicated the polymeric nature of the objects—even a 128-arm star with an arm length of ~60 links did not show signs of colloidal behavior. To further study the properties of multiarm PDMS systems, a series of model 128-arm objects based on a sixth-generation carbosilane dendrimer with different arm lengths were synthesized and studied, and an attempt was made to synthesize a 512-arm PDMS star based on dendrimer G8.

## 2. Results and Discussion

**Scheme 1:**
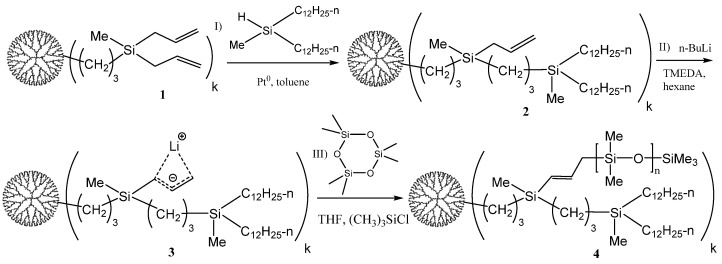
Synthesis of star-shaped PDMS based on carbosilane dendrimer, where k = 32, 128, and 512.

The possibility of obtaining multiarm stars with a known number of arms and their adjustable and known length was determined by the use of the nonequilibrium anionic polymerization of hexamethylcyclotrisiloxane with a regularly structured polylithium dendritic initiator (Scheme 1). The polyfunctional initiator was a derivative of a carbosilane dendrimer with a protective hydrocarbon layer to prevent the intermolecular aggregation of lithium atoms [31].

The shielding outer layer consisting of methyldidodecylsilyl groups was created by the hydrosilylation of didodecylmethylsilane (DDMS) and dendrimer **1**, leaving half of the outer allyl groups unreacted (Figures S1–S4). Based on the synthesized DDMS derivative **2**, we obtained a polylithium anionic polymerization macroinitiator **3** via reaction with n-butyllithium and TMEDA in a hexane solution. The lithiation reaction was monitored by the appearance of lithium carbanion signals on the ^1^H NMR spectra, along with disappearance signals from allyl groups, using diffusion filtration to suppress hexane signals. Based on the absence of signals from allyl groups in the ^1^H NMR spectra (Figure S5), it is fair to say that the conversion of the carbanion formation reaction in the case of DDMS derivatives of fourth- and sixth-generation dendrimers was close to 100%, which allows, within the NMR technique error, one to assert with a high degree of certainty the presence of active lithium centers in the structure of the macroinitiator, in an amount similar to the number of allyl groups in the dendrimer-derived DDMS. Earlier, the GPC technique showed [29] that the organolithium product of lithiation terminated by trimethylchlorosilane is monomodal and has a narrow polydispersity, which proves the complete absence of intermolecular crosslinking reactions.

**Figure 1:**
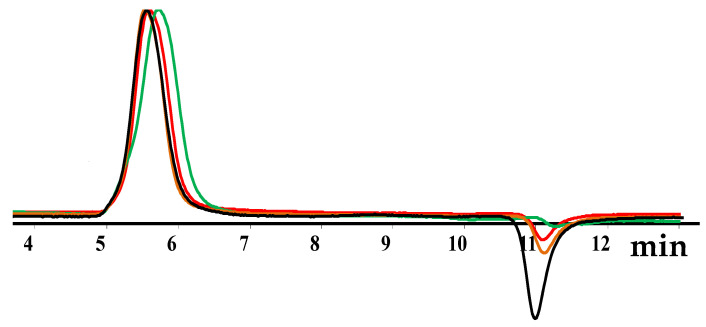
GPC curves of star-shaped PDMSs with number of arms f = 128 and different arm lengths.

The polymerization of hexamethylcyclotrisiloxane using a synthesized polyfunctional initiator with different monomer/initiator ratios (Table S1) yielded a series of 128-arm star-shaped PDMSs, with monomodal narrow-dispersed molecular weight distributions according to GPC data (Figure 1). The presence of a certain number of didodecylsilyl group protons on the branching center in the structure of the synthesized compounds made it possible to calculate the number average molecular weight of the macromolecule and the arm length using the ^1^H NMR spectra data (Figures S6–S9, Formula S1). The results of calculations are presented in Table 1.

In the text, samples of star-shaped polydimethylsiloxanes (PDMS) are designated as St-arms number-arm length, for example, St-128-114.

**Figure 2:**
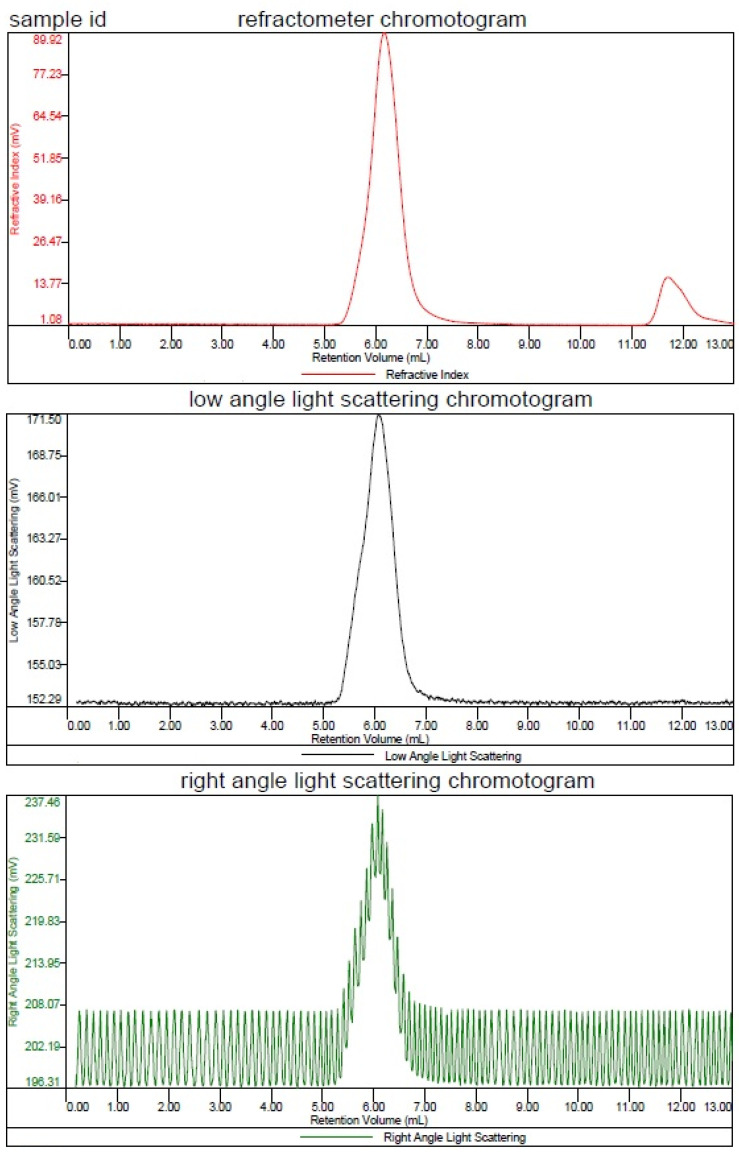
Static light scattering data obtained for St-128-33.

The light scattering method confirmed the monomodality and narrow dispersion of the obtained objects according to the GPC data; as an example, the obtained curves for a specimen St-128-33 are shown in Figure 2.

**Figure 3:**
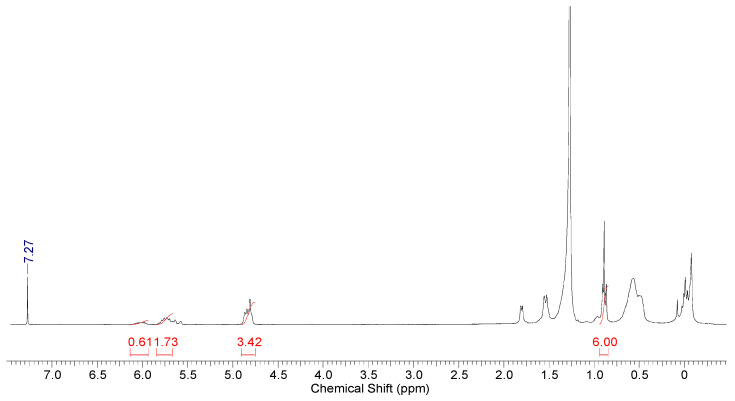
^1^H NMR spectrum of the purified DDMS derivative of the eighth-generation carbosilane dendrimer.

Thus, the synthesis of the 32-arm PDMS star as a comparison object and a series of 128-arm PDMS stars with different arm lengths was successfully carried out; however, the use of this approach for the synthesis of star-shaped 512-arm PDMSs with an eighth-generation carbosilane dendrimer as the initial compound was unsuccessful. While the structure of the initial allyl functional G8 dendrimer, according to GPC and ^1^H NMR spectroscopy data, corresponded to the theoretical one, the reaction of the didodecylmethylsilane hydrosilylation of half of the allyl groups did not yield the target conversion of the hydrosilyl groups above ~60%. Even with the toughening of the reaction conditions and a significant increase in the reaction time, only the increased migration of the double bond was observed (Figure 3).

**Figure 4:**
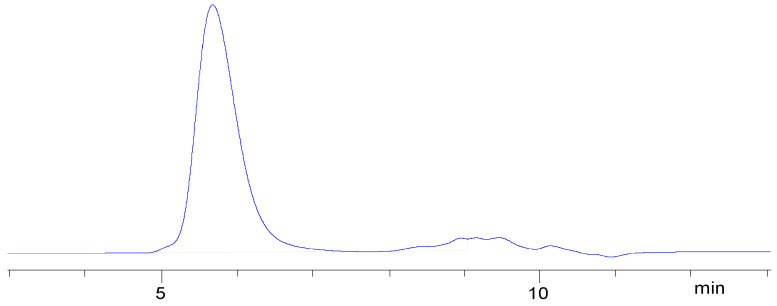
GPC curve of the 8th generation dendrimer DDMS derivative reaction mixture.

The GPC of the reaction mixture showed the presence of a low-molecular-weight fraction, probably as a result of the presence of side reactions of hydrosilanes with the toughening of the process conditions (Figure 4).

It can be assumed that, in the case of a G8 dendrimer, the outer layer is packed so densely that the accessibility of all unsaturated groups is impaired, and the steric factor does not allow the required number of hydrosilyl group additions to occur.

The lithiation reaction also proceeded slowly and not to the same high conversion as in the case of dendrimer G6. The introduction of an additional amount of n-BuLi and the continuation of the reaction for 92 h did not result in a target conversion of allyl groups, which noticeably differentiates the processes on the eighth-generation dendrimer from similar reactions using previous objects of this series with less dense outer layers (Figure S10).

Thus, it was found that the processes on dendrimer G8, both the silylation of half of the allyl groups and lithiation of the remaining ones, were much more difficult, and they proceeded to a lower depth than in previous generations. In this case, the tougher reaction conditions, as well as an increase in their duration, did not lead to an increase in the conversion of functionalities, which indicates the presence of inaccessible groups, apparently due to steric hindrances in the especially dense outer sphere of the eighth-generation dendrimer, as there were no other differences from processes on the previous generations. Quite naturally, the use of such an initiator, which possessed unequally active centers from 500 to 700 in the anionic polymerization of hexamethylcyclotrisiloxane, led to the formation of a star-shaped product with obvious polydispersity both in the number of arms and in their length. Product fractionation in a preparative chromatograph and fraction composition analysis by ^1^H NMR spectroscopy showed that the content of the dimethylsiloxane component was ~10 times as high in the low-molecular-weight fraction compared to the high-molecular-weight fraction.

Thus, it can be stated that the sixth-generation carbosilane dendrimer is a kind of threshold, the last object in the series of carbosilane dendrimers with an outer layer density sufficient for full accessibility and equivalence of all terminal functional groups. It should be noted that, already in this case, the silylation processes were slower and under tougher conditions than in the previous generations; however, as a result, their target conversion was achieved. The structure of the eighth-generation carbosilane dendrimer, apparently due to steric hindrances and the appearance of inaccessible groups, did not allow target conversion of the terminal functionalities, which prevented the obtaining of a regularly structured polyfunctional macroinitiator based on it.

Earlier, we showed [31] that low-arm star-shaped PDMSs with f = 8 and 32 were Newtonian fluids, similar to linear PDMS, but an increase in the number of arms (f = 128 in our case) led to the appearance of pseudoplastic properties. The obtained flow curves of the synthesized series of 128-arm PDMS showed (Figures S12–S15) that, while the specimens with an arm length of up to 100 dimethylsiloxane units were shear thinning liquids, an increase in the arm length to n = 114 again demonstrates a flow pattern close to Newtonian, that is, the rheological properties were again determined by the behavior of the lateral PDMS arms. Moreover, based on repeated measurements of the flow curve upon cooling to room temperature after heating, it can be seen that the original curve at 20° did not coincide with the curve obtained after cooling the specimens, which may indicate the presence of slow relaxation processes in the star-shaped PDMS specimens.

Based on the obtained flow curves, viscous flow activation energy values were calculated for all 128-arm star-shaped PDMSs using the Arrhenius Equation (1):

η = Ae^Ea/RT^ (1)

The values obtained in this series insignificantly differ from the E_act_ of linear PDMSs [32], and at a large arm length n = 114, their values completely coincide, which characterizes even such multiarm high-density structures within the considered arm lengths as polymeric objects rather than colloidal particles (Table 1).

**Figure 5:**
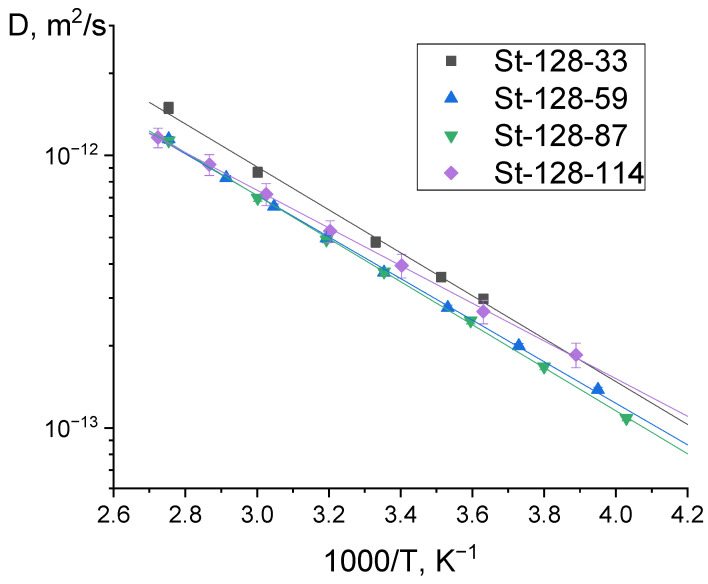
Dependence of the average self-diffusion coefficient of 128-arm PDMSs with different arm lengths on temperature.

A structural investigation of the synthesized series of specimens by means of PFG NMR in diluted solutions of toluene confirmed their narrow dispersion. The determined self-diffusion E_a_ of 128-arm PDMS stars in melts are presented in Table 1. Diffusion attenuation was obtained as the dependence of the stimulated echo amplitude (A) on the amplitude of the magnetic field gradient (g). The self-diffusion coefficient (SDC) was determined from the diffusion attenuation (Equation (2):, according to the expression [33]:

(2) $$A=A_{0}\exp\left(-\gamma^{2}g^{2}\delta^{2}t_{d}D\right)\;(*)$$

where γ is the gyromagnetic ratio, δ is the gradient pulse duration, t_d_ = (Δ−δ/3) is the effective diffusion time, Δ is the interval between the gradient pulses, and D is the self-diffusion coefficient. For diluted star-shaped PDMS solutions in toluene, the diffusion attenuations had an exponential form (2), which confirms the narrow dispersion of the molecular weight distribution. The SDC values were of the order of 1 × 10^−10^ m^2^/s. A complex nonexponential attenuation form was observed for the 128-arm star-shaped PDMS melts. The gradient pulse duration δ and the diffusion time t_d_ were 3 and 99 ms, respectively, in the experiments used to determine the activation energy of the self-diffusion (Figure 5). The attenuations were approximated by two terms of the form (2) with relative contributions p_1_ and p_2_ (p_1_ + p_2_ = 1), from which an average SDC was determined Equation (3):

(3) $$D=p_{1}D_{1}+p_{2}D_{2}$$

The values of p_1_ and p_2_ were selected individually for each specimen but were kept constant at different temperatures. Such an approach allows for a good approximation of at least a fifty-fold decay of the stimulated echo amplitude. The p_1_ values corresponding to the higher self-diffusion coefficient were in the range of 0.3–0.7. The dependence of the average coefficient on temperature is shown in Figure 5. The self-diffusion activation energy values were determined using approximation by Arrhenius dependences of the form Equation (4) :

D = D_0_e^−Ea/RT^ (4)

in the corresponding linear coordinates. The results are shown by solid lines in Figure 5. The greatest differences between D_1_ and D_2_ SDCs, as well as between the values of p_1_ and p_2_, were observed for the specimen with the highest molecular weight (St-128-114). For this sample, a series of experiments with varying diffusion time t_d_ was performed at 25 °C. Changes in the shape of diffusion attenuation were not observed for t_d_ in the range of 100–500 ms. Thus, the restricted in-cage motion, which is characteristic of the behavior of colloidal suspensions [26], was not observed on the considered timescale. On the other hand, the appearance of nonexponential diffusion attenuations in melts contrasted the exponential attenuations in diluted solutions, indicating the presence of dynamical heterogeneities. On the whole, the activation energies of multiarm star-shaped PDMSs were close to each other. These values are slightly lower than the viscous flow activation energies and are also lower than the value of ~18.8 kJ/mol determined for the self-diffusion of linear PDMSs [34].

The synthesized star-shaped polymers were studied via DSC.

**Figure 6:**
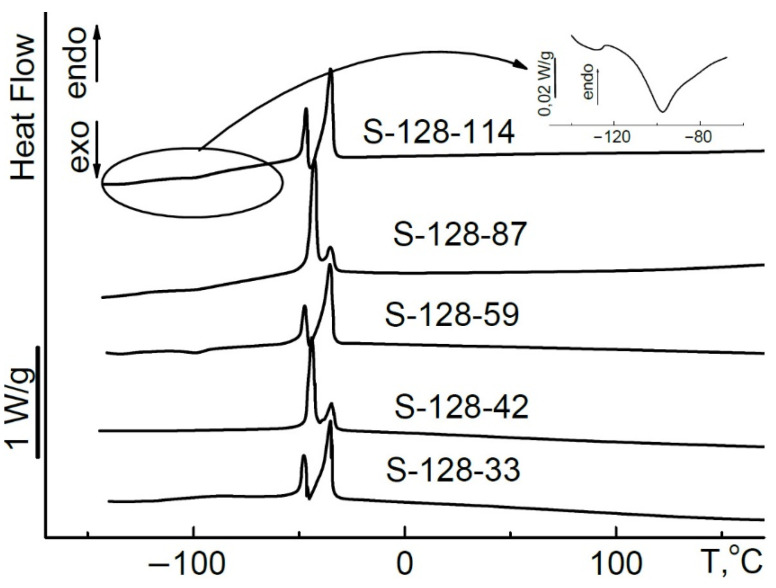
DSC curves of star-shaped PDMSs with different arm lengths.

It was found that the DSC curves (Figure 6) showed thermal effects characteristic of a linear PDMS [35]. The jump in heat capacity at the glass transition temperature was expressed very weakly, as well as the exothermic cold crystallization effect (Table 2). The glass transition temperature of the synthesized stars was generally lower than that of a linear PDMS, similar to those observed in other star-shaped systems [36,37]. The melting peak of the crystalline phase was bimodal. The heat of fusion was lower than that observed for a linear PDMS with a comparable molecular weight, as was previously observed [22,23,24]. Up to a temperature of 170 °C, no heat effects were observed above the melting of the PDMS crystalline phase.

**Figure 7:**
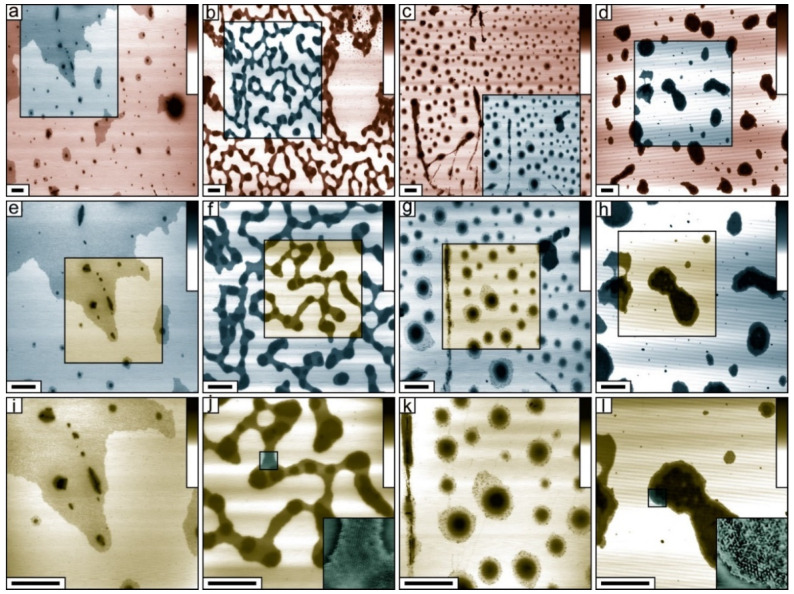
AFM results. From left to right: St-32-230 (G4 dendrimer as object of comparison) (**a**,**e**,**i**); St-128-33 (G6 dendrimer) (**b**,**f**,**j**); St-128-114 (G6 dendrimer) (**c**,**g**,**k**); St ~190-10 (G8 dendrimer) (**d**,**h**,**l**). Height scale, 30 nm. Scale line, 500 nm. Frame sizes in rows from top to bottom are 8 × 8 µm (**a**–**d**), 4 × 4 µm (**e**–**h**), and 2 × 2 µm (**i**–**l**). The inset images show a size of 190 × 190 nm (St-128-33 j, St ~190-10 l). The areas in rectangles have been rescanned and are shown in the figures below on a larger scale.

The results of examining the properties of multiarm stars have led to conflicting outcomes. On the one hand, we have received answers to key questions as to which system these objects are closer to—the colloidal or polymeric one. On the other hand, the DLS and GPC data indicated the monodisperse nature of the objects obtained, but the study of self-diffusion in melt indicated the presence of objects with different diffusion coefficients, while, in dilute solutions, we also see monodisperse objects by self-diffusion measurements. The impeccability of the synthetic approach also turns out to have its limits. In addition, while side processes during the synthesis of multiarm stars based on an eighth-generation dendrimer were expected, certain doubts arose: they were possibly also observed to a lesser extent when using lower generations. Indeed, in other cases, the formation of inhomogeneities was also reported during the drying of star specimens. Most of these doubts were dispelled at the stage of structure identification. As was already noted, specimens of sixth-generation dendrimers of different consistency always gave an identical monomodal GPC pattern and equally adequate NMR spectra. The unexpected diffusion coefficient results were attributed to the increased aggregation tendency of multiarm objects. Similar to dendrimers [38], while dried, multiarm stars form metastable aggregates that are stable under normal conditions. They become completely destroyed when dissolved; therefore, we see monomodal narrowly dispersed signals on GPC or DLS. The results of examining these objects by atomic force microscopy techniques gave rich food for thought (Figure 7).

The AFM results showed that small stars (St-32-230, G4 dendrimer) are prone to aggregation: we saw a continuous layer of aggregated stars and individual aggregates in the figures in the first vertical column (a, e, i) with a sequential increase in resolution. The transition to objects obtained on the sixth-generation dendrimer (St-128-33 and St-128-114) led to a change in the aggregate nature. In the case of long arms (c, g, k), they were presented in the form of dense multilayer spherical objects and, at the same time, individual particles were observed, which, in terms of their size, can be attributed to single stars. Their diffusion characteristics were significantly lower than those of the lighter analogs from the first column, and in the process of rapid contraction of the solution, some of them “stray from the herd”. Thus, the AFM data to a certain extent correlate with the results of self-diffusion studies.

However, of course, the most spectacular shots were obtained on the most unsuccessful specimen with a G8 dendrimer as a nucleus (St ~190-10). In this case, as described above, all the processes did not follow the general pattern; there was a cross-transition of the chain to the neighboring arms, and free linear PDMS oligomers and compacted systems were formed, which, of course, can be attributed to multiarm stars only conditionally. On the other hand, thanks to the stability of the core and the compaction of the shells, we see that after fractionation, these objects were prone to the formation of ordered layers with a crystal-like structure. The period of the visualized two-dimensional lattice (shown in the inserts) was 9.2 ± 0.6 nm for specimen St ~ 190-10 (G8 dendrimer, compacted siloxane shell) and 7.0 ± 0.4 nm for specimen St-128-33 (G6 dendrimer). Obviously, these numbers determine the lateral size of the corresponding individual polymeric object in the closely packed layer.

## 3. Materials and Methods

In this work, the following reagents and excipients were used: the carbosilane dendrimers G4, G6, and G8 with allyl functional groups in the shell, previously obtained using the technique from [39]; methyldichlorosilane 97% distilled with a reflux condenser manufactured by ABCR ((Karlsruhe, Germany); distilled allyl chloride 98% manufactured by Acros (Geel, Belgium); hexane 97.76% manufactured by Ruskhim.ru (Moscow, Russia), dried over calcium hydride and 3 Å molecular sieves; analytical-grade toluene manufactured by Khimpromtorg (Reutov, Russia), dried over sodium with benzophenone and 3 Å molecular sieves; tetrahydrofuran (THF) 99.8% manufactured by Ruskhim.ru, dried over sodium with benzophenone and 3 Å molecular sieves; platinum(0)-1,3-divinyl,1,1,3,3-tetramethyldisiloxane complex–2% solution in xylene (Karstedt’s catalyst, Sigma-Aldrich, St. Louis, Missouri, MO, USA); 1-bromododecane 98% manufactured by Acros, magnesium shavings 99% manufactured by Fluka (Sigma-Aldrich, St. Louis, Missouri, MO, USA); hexamethylcyclotrisiloxane (D_3_) 95% manufactured by ABCR, dried and distilled over calcium hydride; n-butyllithium 1.6 M solution in hexane manufactured by Acros; tetramethylethylenediamine (TMEDA) (99%, Acros), dried over 3 Å molecular sieves; petroleum ether distilled on a rotary evaporator manufactured by Ruskhim.ru; silica gel manufactured by Khimmed (Moscow, Russia), 0.040–0.063 mm; anhydrous sodium sulfate manufactured by Komponent-reaktiv (Moscow, Russia); saturated ammonium chloride solution manufactured by MCD (Moscow, Russia).

GPC analysis was carried out on the following chromatographic system: a STAYER s.2 high-pressure pump (Akvilon, Moscow, Russia), a Smartline RI 2300 refractometer, and a JETSTREAM 2 PLUS thermostat (KNAUER, Berlin, Germany). The thermostat temperature was 40 °C (±0.1 °C). The eluents were THF and toluene +2% THF; the flow rate was 1.0 mL/min. Columns 300 × 7.8 mm, Phenogel sorbent (Phenomenex, Los Angeles, CA, USA), 5 μm with pores ranging from 10^3^ to 10^5^ Å.

^1^H NMR spectra were recorded on a Bruker WP250 SY spectrometer (Billerica, Massachusetts, MA, USA), CDCl_3_ solvent, as well as on a BrukerAvance AV300 spectrometer (Billerica, Massachusetts, MA, USA), tetramethyl silane external standard, n-hexane and CDCl_3_ solvent. The spectra were processed on a computer while using the ACD/ChemSketch software, version 2020.1.2, Advanced Chemistry Development, Inc., (Toronto, ON, Canada).

Self-diffusion studies were carried out via pulsed magnetic field gradient NMR (PFG NMR),) on ^1^H nuclei using the stimulated echo technique [32] on a Bruker Avance III–400 spectrometer (Billerica, Massachusetts, MA, USA). The spectrometer is equipped with a gradient system that allows one to obtain magnetic field gradient pulses with a maximum amplitude of 30 T/m. Diffusion attenuations were obtained as a function of the gradient amplitude at constant time intervals in the pulse sequence. The temperature was controlled within ±0.2 °C. The samples of neat star-shaped PDMS (melts) and 0.9%-by-weight solution in toluene (diluted solutions) were investigated.

Rheological characteristics were measured on an Anton Paar MCR 302 rheometer (Graz Austria) in constant shear rate mode with a cone-plane measuring unit (d = 25 mm).

Differential scanning calorimetry (DSC) studies were carried out on a DSC-822e instrument (Mettler-Toledo, Greifensee, Switzerland) in an argon atmosphere at a heating rate of 10 °C/min.

AFM experiments were carried out on a multimode scanning probe microscope with a NanoScope-IIIa controller and an MMAFM-2 scanning head in tapping mode (Digital Instruments, Santa Barbara, CA, USA). We used silicon cantilevers manufactured by Nanoworld ( Neuchatel, Switzerland) with a resonant frequency of about 300 kHz. The recording density was 512 × 512 points at a horizontal-sweep frequency of 1 Hz. The recorded images were analyzed using the Nanoscope (Digital Instruments, Tonavanda, New York, NY, USA) and Femtoscan (ATC, Moscow, Russia) software. For examination, 1 μL of polymer object solution in hexane at a concentration of c = 1 g/L was applied to the surface of freshly cleaved mica and was dried in air.

## 4. Experimental Part

The polylithium initiator was synthesized using 4th, 6th, and 8th generation carbosilane dendrimers with an outer hydrocarbon layer according to the technique described in [4].

Multiarm star-shaped polydimethylsiloxanes were obtained by the anionic polymerization of hexamethylcyclotrisiloxane using a polylithium initiator in an amount corresponding to the given arm length in a 10% hexane solution with a THF polymerization promoter in a glove box under an inert atmosphere. The polymerization time ranged from 14 to 24 h, and the product was terminated afterward with trimethylchlorosilane. The resulting polymer was purified by extraction, filtration, and preparative gel permeation chromatography.

## 5. Conclusions

A series of 128-arm star-shaped PDMSs with arm lengths from 33 to 114 siloxane units were obtained using the core-first approach with a branching center based on carbosilane dendrimer G6 having a didodecylmethylsilyl layer that prevents the intermolecular aggregation of lithium atoms. It was found that the G6 dendrimer is the last generation where the packing density of the outer layer allows the existence of equally accessible and equally active terminal functional groups. When carrying out similar reactions of the terminal groups of a carbosilane dendrimer G8, the processes proceed with a noticeably lower conversion, and tougher reaction conditions, as well as an increase in their duration, lead to a number of side processes, such as the transition of the chain to neighboring arms, and unequal development of the polymerization process from the beginning. These results give us reason to say that the proposed approach for the formation of multiarm stars has limits for the formation of regular structures, and the eight generation is outside of this limit.

The rheology of the synthesized series almost completely coincides with the behavior of a linear PDMS with respect to viscous flow E_act_ when their viscosity characteristics differ by several orders of magnitude. In this case, specimens of 128-arm star-shaped PDMS with short arms, which are shear thinning liquids, fall out of the state of Newtonian fluids, usual for PDMS. All this characterizes even such multiarm high-density structures within the considered arm lengths as polymeric objects rather than colloidal particles. DSC data show the presence of glass transition and crystallization processes in the synthesized objects, completely identical to a linear PDMS.

## Figures and Tables

**Table 1:** Characteristics of the obtained star-shaped 128-arm PDMSs.

Sample	PDI	MW (According to ^1^H NMR)	E_a_ of the Viscous Flow, kJ/mol	Viscosity (at 20 °C, Shear Rate 10 s^−1^)	E_a_, kJ/mol (Self-diffusion, Melt)	D, 10^−13^ m^2^/s (20 °C, Melt)	D, 10^−11^ m^2^/s (25 °C, 0.9% Solution in Toluene)	Hydrodynamic Radius R_h_, nm (25 °C, 0.9% Solution in Toluene)	E_a_, kJ/mol (Self-diffusion, 0.9% Solution in Toluene)
St-128-33	1.12	402,700	17.7	4.04	15.1	4.32	8.97	4.06	9.58
St-128-59	1.19	639,500	18.5	4.39	14.6	3.47	8.97	4.42	8.78
St-128-87	1.07	914,200	18.5	3.11	15.1	3.37	9.37	4.24	9.29
St-28-114	1.07	1,169,900	16.1	2.56	13.2	3.87	8.59	4.62	8.59

**Table 2:** Thermal characteristics of 128-arm PDMSs with various arm lengths.

Sample	T_g_, °C	T_cc_, °C	∆H_cc_, J/g	T_m_, °C	∆H_m_, J/g
St-128-33	−126	−97	0.1	−48/−35	27.8
St-128-59	−128	−99	2.0	−47/−36	27.5
St-128-87	−121	−97	4.0	−42/−36	30.5
St-128-114	−125	−98	3.3	−49/−35	31.7

## Data Availability

The data presented in this study are available in supplementary material.

## Supplementary Materials

The following are available online. Figure S1: GPC curve of DDMS-derivative of carbosilane dendrimer of 4th generation, Figure S2: GPC curve of DDMS-derivative of carbosilane dendrimer of 6th generation, Figure S3: 1H NMR spectrum of DDMS-derivative of carbosilane dendrimer of 4th generation, Figure S4: 1H NMR spectrum of DDMS-derivative of carbosilane dendrimer of 6th generation, Figure S5: 1H NMR spectra recorded during lithiation of the 6th generation DDMS dendrimers without (left) and with (right) using diffusion filtration after 20 (a) and 44 h (b), Figure S6: 1H NMR spectrum of St-128-33, Figure S7: 1H NMR spectrum of St-128-59, Figure S8: 1H NMR spectrum of St-128-87, Figure S9: 1H NMR spectrum of St-128-114, Figure S10: 1H NMR spectra of the product of lithiation with n-butyllithium of the DDMS-derivative of carbosilane dendrimer G8 using diffusion filtration after 68 (1) and 92 h (2) since the start of the reaction, Figure S11: GPC curve of DDMS-derivative of the carbosilane dendrimer G8, Figure S12: Flow curves of St-128-33 at temperatures from 20 to 120 °C and re-measured at 20 °C after cooling from 120 °C, Figure S13: Flow curves of St-128-59 at temperatures from 20 to 120 °C and re-measured at 20 °C after cooling from 120 °C, Figure S14: Flow curves of St-128-87 at temperatures from 20 to 120 °C and re-measured at 20 °C after cooling from 120 °C, Figure S15: Flow curves of St-128-114 at temperatures from 20 to 120 °C and re-measured at 20 °C after cooling from 120 °C, Table S1: Polymerization parameters of 128-arm PDMS.

## Sample Availability

Samples of the compounds are available from the authors.
