# Supplementary material for: Multiarm Star-Shaped Polydimethylsiloxanes with a Dendritic Branching Center
Source: Molecules. 2021 May 29;26(11):3280. doi: 10.3390/molecules26113280 (PMC8199136; doi:10.3390/molecules26113280)
Supplement: Supplementary file 1 [file molecules-26-03280-s001.zip › molecules-1229921-supplementary.pdf]

molecules

MDPI

Article

Multiarm Star-shaped Polydimethylsiloxanes with A Dendritic Branching Center

Pavel A. Tikhonov [1], Nataliya G. Vasilenko [1,*], Marat O. Gallyamov [2,3], Georgii V. Cherkaev [1], Viktor G. Vasil'ev [2], Nina V. Demchenko [1], Mikhail I. Buzin [2], Sergey G. Vasil'ev [4] and Aziz M. Muzafarov [1,2]

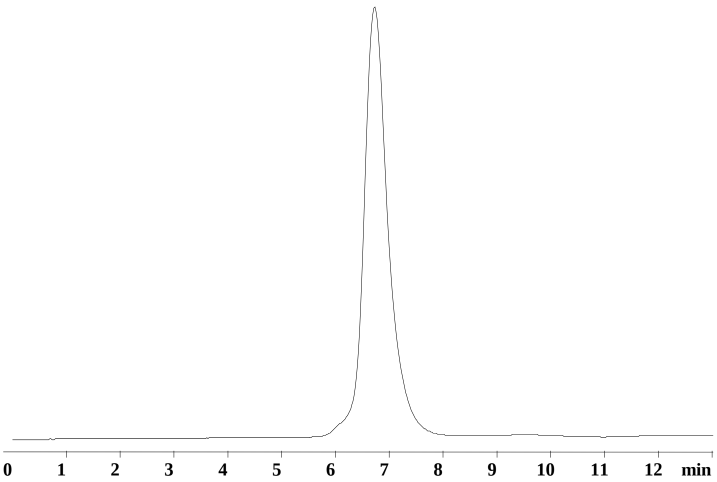

Figure S1. GPC curve of DDMS-derivative of carbosilane dendrimer of 4th generation.

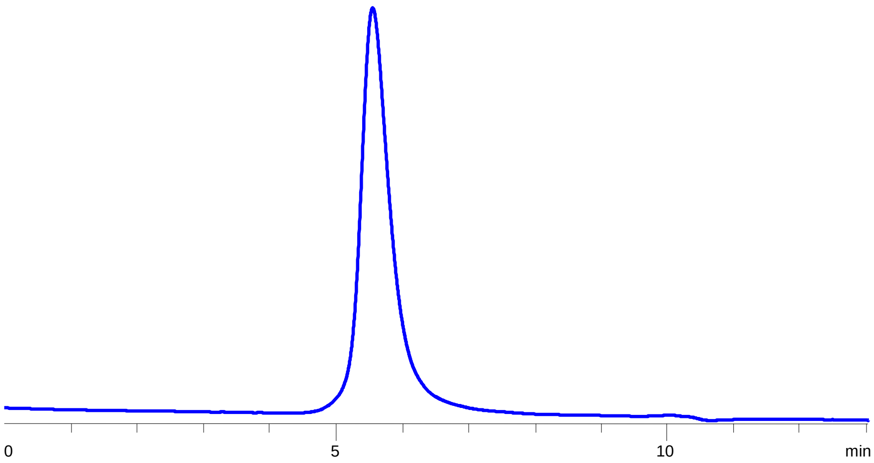

Figure S2. GPC curve of DDMS-derivative of carbosilane dendrimer of 6th generation.

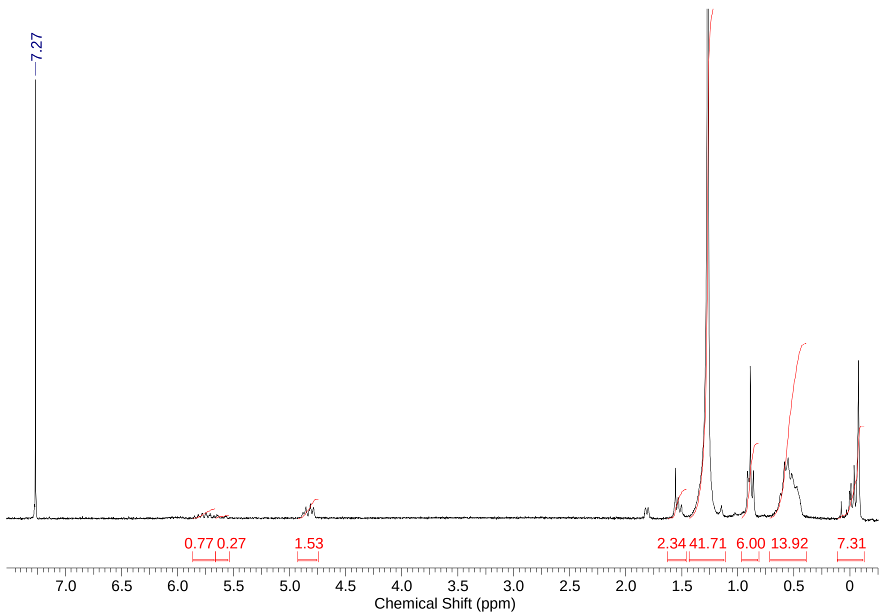

Figure S3. ^{1}H NMR spectrum of DDMS-derivative of carbosilane dendrimer of 4th generation: (chloroform - d, 7.27 м.д.) -0.04 (м, 69, $C\underline{H}_3$-Si-); 0.55 (м, 124, -$C\underline{H}_2$-Si); 0.89 (m, 48, -CH_2-CH_2-$C\underline{H}_3$); 1.27 (м, 366, -CH_2-$C\underline{H}_2$-CH_2-); 1.54 (м, 16, -$C\underline{H}_2$-CH=CH_2); 4.82 (м, 16, -CH_2-CH=$C\underline{H}_2$), 5.76 (м, 8, -CH_2-$C\underline{H}$=CH_2).

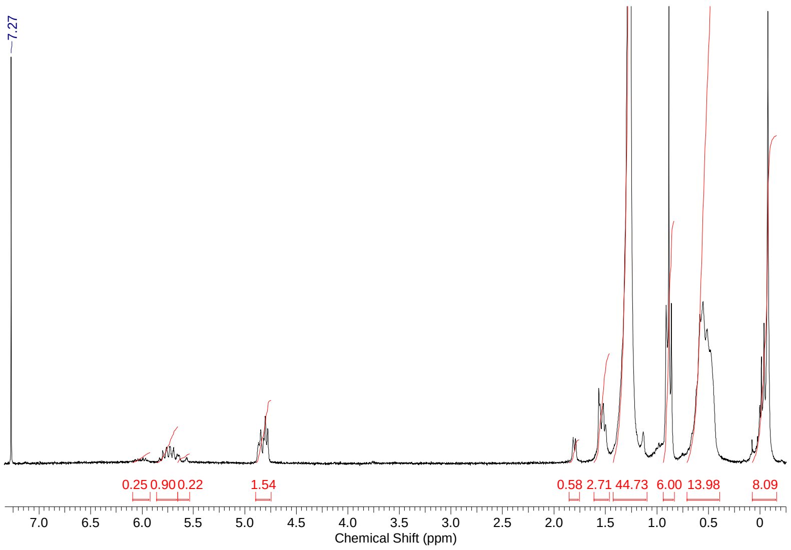

Figure S4. ^{1}H NMR spectrum of DDMS-derivative of carbosilane dendrimer of 6th generation: (chloroform - d, 7.27 м.д.) -0.04 (м, 285, $C\underline{H}_3$-Si-); 0.55 (м, 508, -$C\underline{H}_2$-Si); 0.89 (m, 192, -CH_2-CH_2-$C\underline{H}_3$); 1.27 (м, 1470, -CH_2-$C\underline{H}_2$-CH_2-); 1.54 (м, 64, -$C\underline{H}_2$-CH=CH_2); 4.82 (м, 64, -CH_2-CH=$C\underline{H}_2$), 5.76 (м, 32, -CH_2-$C\underline{H}$=CH_2), signals indicating migration of a double bond in allyl groups: 1.8 (д, 27, $C\underline{H}_3$-CH=CH-); 5.61 (д, 9, CH_3-CH=$C\underline{H}$-); 6.02 (м, 9, CH_3-$C\underline{H}$=CH-).

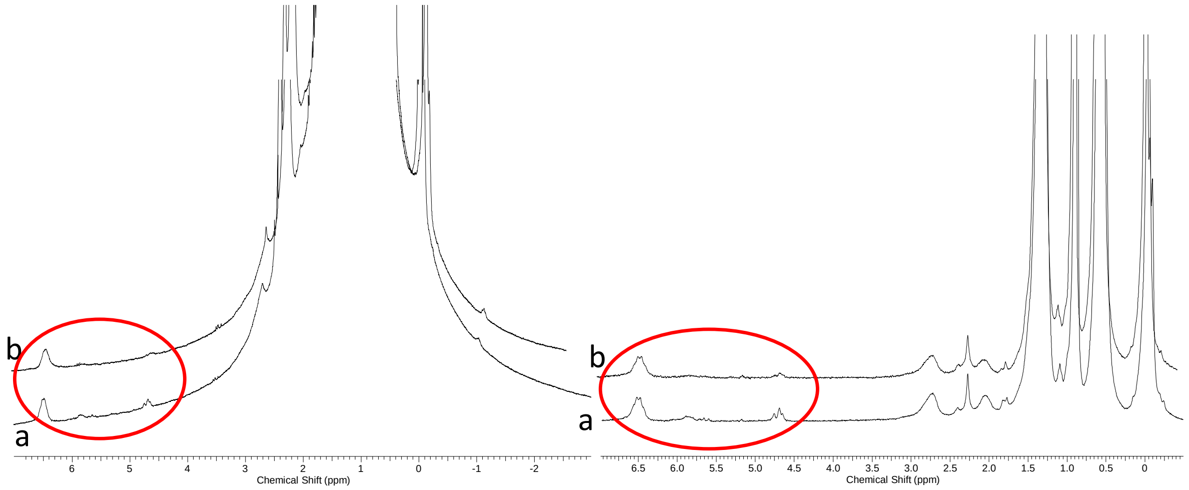

Figure S5. ^{1}H NMR spectra recorded during lithiation of the 6th generation DDMS dendrimers without (left) and with (right) using diffusion filtration after 20 (a) and 44 (b) hours.

Table S1. Polymerization parameters of 128-arm PDMS.

Sample	(G_6DDMS)Li/D_3 ratio	Time of polymerization, h	Yield, %
St-128-33	1/2554.8	24	67
St-128-59	1/4268.6	14	55
St-128-87	1/4266.7	16	43
St-128-114	1/5120.2	23	44

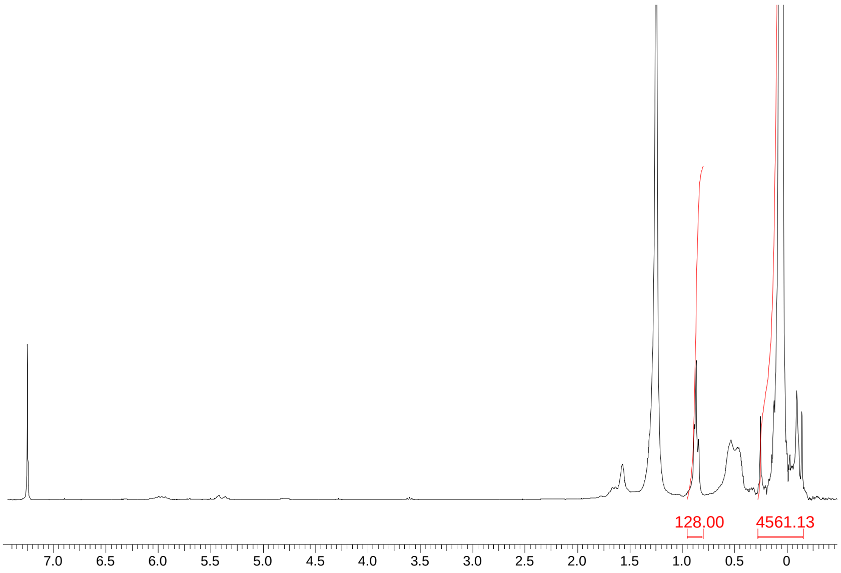

Figure S6. ^{1}H NMR spectrum of St-128-33: (chloroform -d, 7.27 м.д.) 0.08, (м, 6656, $C\underline{H}_3$-Si); 0.55 (м, 352, -$C\underline{H}_2$-Si); 0.89 (m, 192, -CH_2-CH_2-$C\underline{H}_3$); 1.27 (м, 1082, -CH_2-$C\underline{H}_2$-CH_2-), 1.55 (с, 76, -Si-CH_2-$C\underline{H}_2$- и -Si-CH=CH-$C\underline{H}_2$-), 5.42 (д, 12, -Si-C$\underline{H}$=CH-CH_2-), 6.02 (м, 12, -Si-CH=C$\underline{H}$-CH_2-).

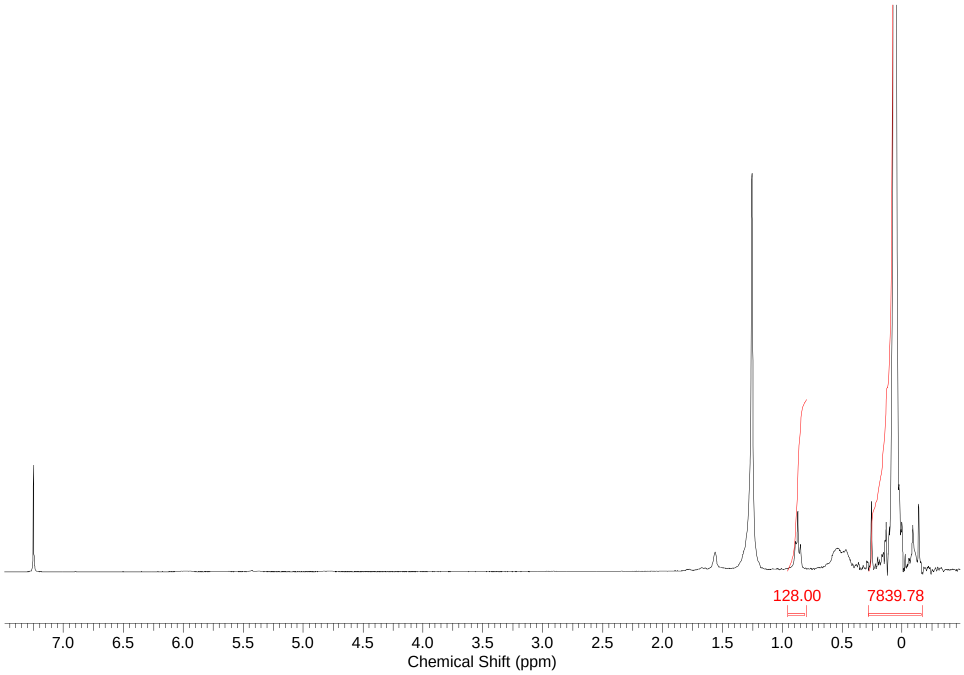

Figure S7. ^{1}H NMR spectrum of St-128-59: (chloroform -d, 7.27 м.д.) 0.08, (м, 11812, $C\underline{H}_3$-Si); 0.55 (м, 326, -$C\underline{H}_2$-Si); 0.89 (m, 192, -CH_2-CH_2-$C\underline{H}_3$); 1.27 (м, 1010, -CH_2-$C\underline{H}_2$-CH_2-), 1.55 (с, 90, -Si-CH_2-$C\underline{H}_2$- и -Si-CH=CH-$C\underline{H}_2$-), 5.42 (д, 8, -Si-C$\underline{H}$=CH-CH_2-), 6.02 (м, 10, -Si-CH=C$\underline{H}$-CH_2-).

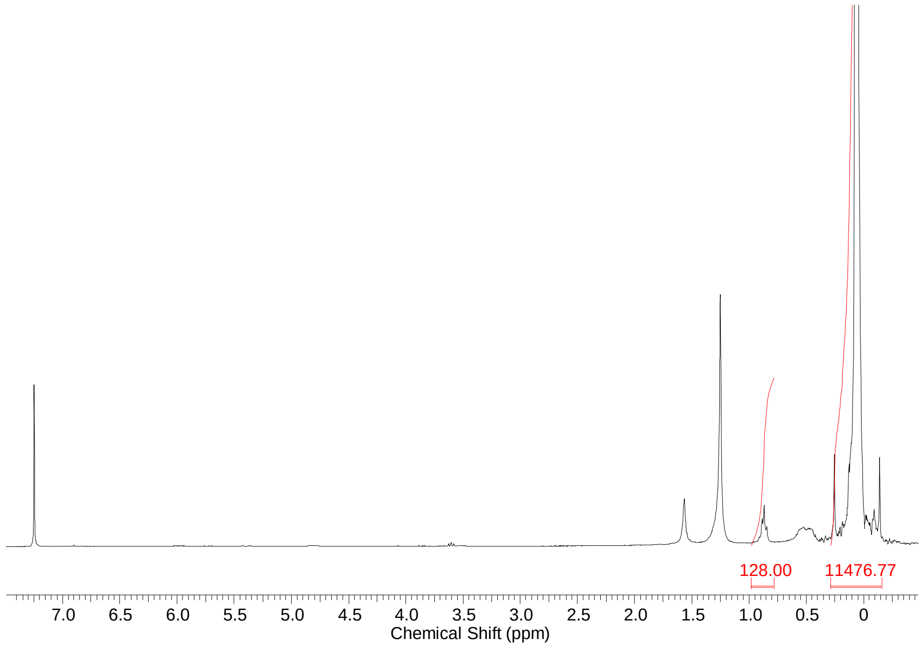

Figure S8. ^{1}*H NMR spectrum of St-128-87: (chloroform -d, 7.27 м.д.): 0.08, (м, 17144,* $C\underline{H}_3$*-Si); 0.55 (м, 294,* $-C\underline{H}_2$*-Si); 0.89 (m, 192,* $-CH_2-CH_2-C\underline{H}_3$*); 1.27 (м, 800,* $-CH_2-C\underline{H}_2-CH_2-$*), 1.55 (c, 180,* $-Si-CH_2-C\underline{H}_2-$ *и* $-Si-CH=CH-C\underline{H}_2-$*), 5.42 (∂, 2,* $-Si-C\underline{H}=CH-CH_2-$*), 6.02 (м, 6,* $-Si-CH=C\underline{H}-CH_2-$*).*

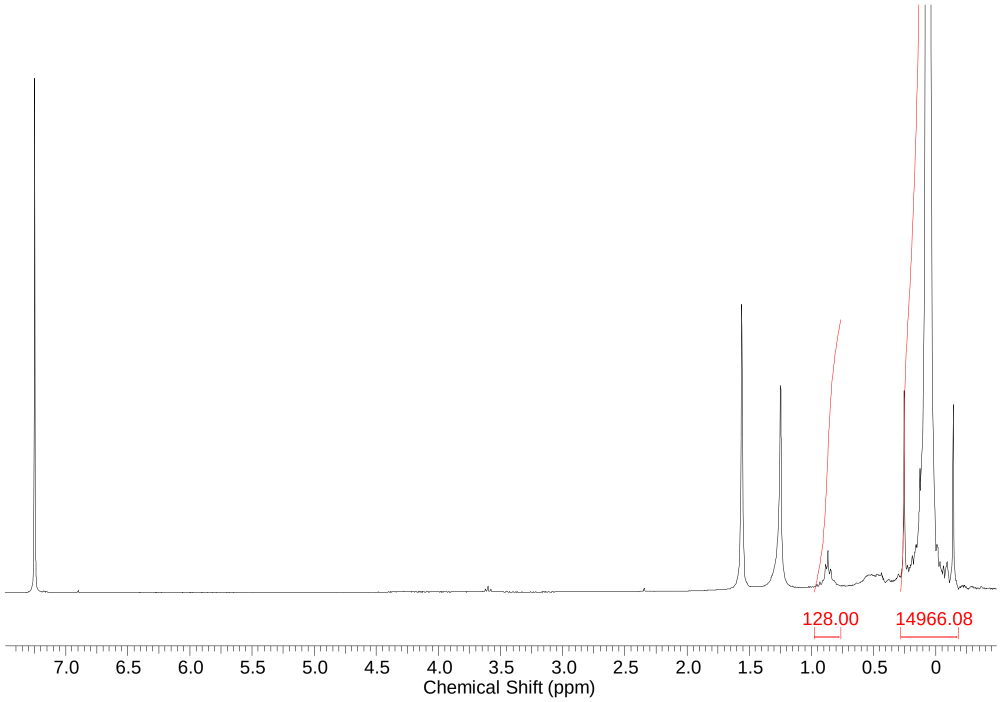

Figure S9. ^{1}*H NMR spectrum of St-128-114: (chloroform-d, 7.27 м.д.) 0.08, (м, 23960,* $C\underline{H}_3$*-Si); 0.55 (м, 210,* $-C\underline{H}_2$*-Si); 0.89 (m, 192,* $-CH_2-CH_2-C\underline{H}_3$*); 1.27 (м, 486,* $-CH_2-C\underline{H}_2-CH_2-$*), 1.55 (c, 396,* $-Si-CH_2-C\underline{H}_2-$ *и* $-Si-CH=CH-C\underline{H}_2-$*).*

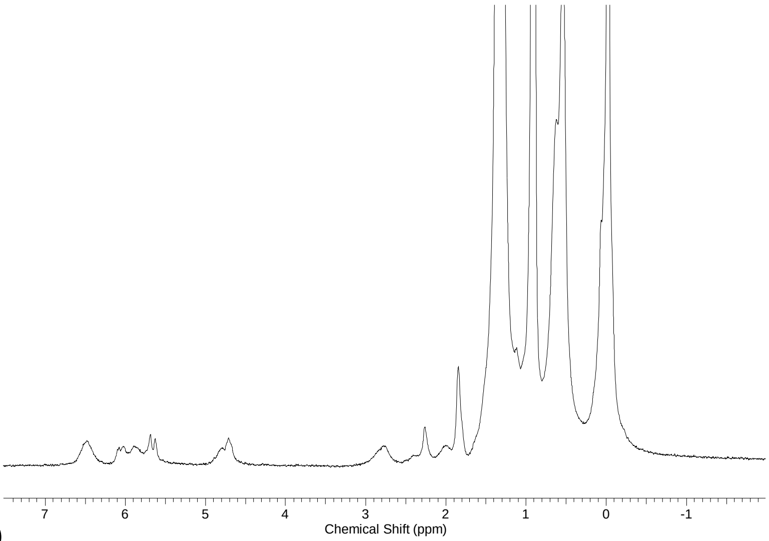

1)

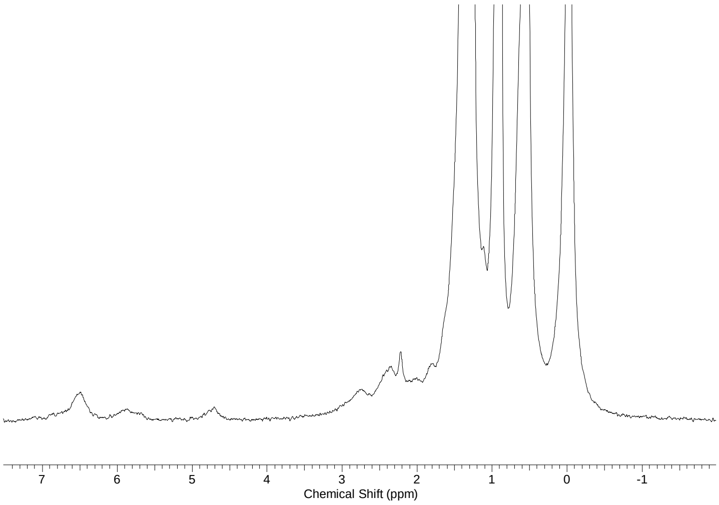

2)

Figure S10. ¹H NMR spectra of the product of lithiation with n-butyllithium of the DDMS-derivative of carbosilane dendrimer G8 using diffusion filtration after 68 (1) and 92 hours (2) since the start of the reaction.

Formula S1. Calculation of the arm length for 128-arm PDMS:

$$L_{f((CH3)2SiO\ units)} = \frac{(I(CH3-Si-)\ -\ 128*9(\text{terminal TMS}-\text{groups})\ -\ (2\hat{}6-4)*3(\text{CH3 from dendrimer core})-128*3(\text{CH3 from DDMS groups}))}{128(\text{arms})*6(\text{protons in (CH3)2SiO units})}$$

$$= \frac{(I(CH_3-Si-)\ -\ 2292)}{768}$$

(at the value of the integral intensity of the signal of CH_3-groups in DDMS substituents 0.9 ppm = 768)

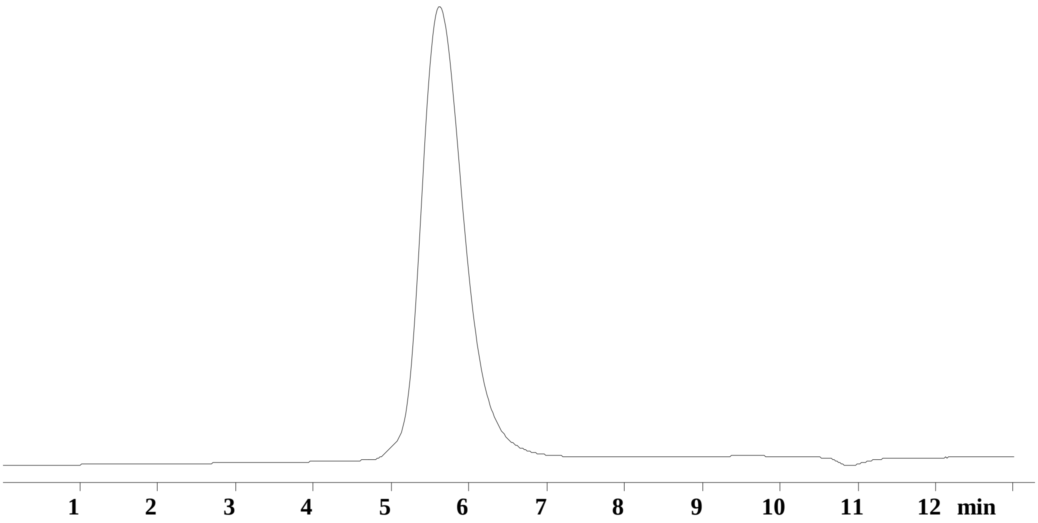

Figure S11. GPC curve of DDMS-derivative of the carbosilane dendrimer G8.

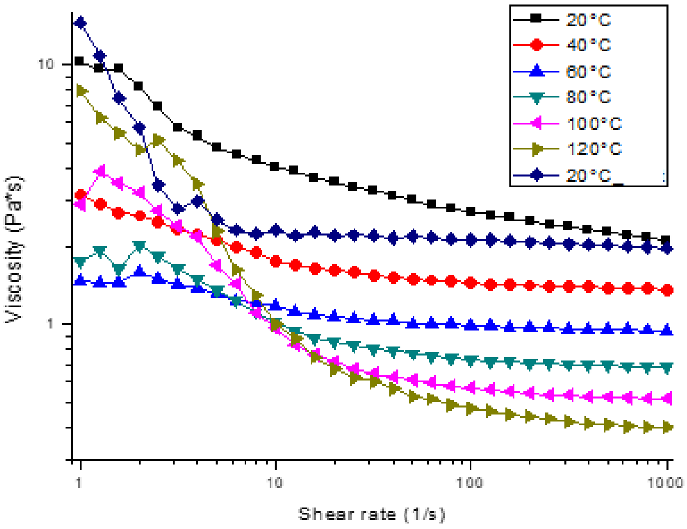

Figure S12. Flow curves of St-128-33 at temperatures from 20 to 120 °C and re-measured at 20 °C after cooling from 120 °C

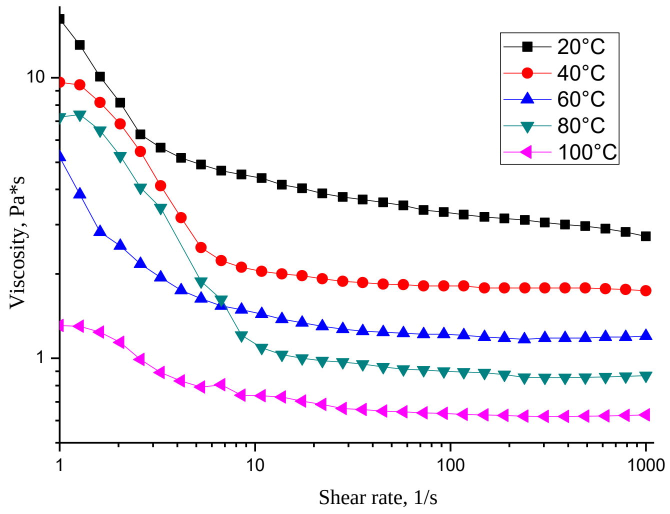

Figure S13. Flow curves of St-128-59 at temperatures from 20 to 120 °C and re-measured at 20 °C after cooling from 120 °C

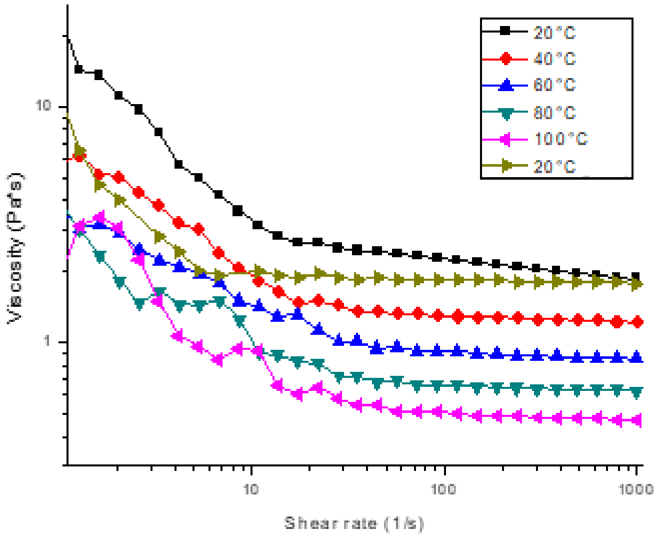

Figure S14. Flow curves of St-128-87 at temperatures from 20 to 120 °C and re-measured at 20 °C after cooling from 120 °C

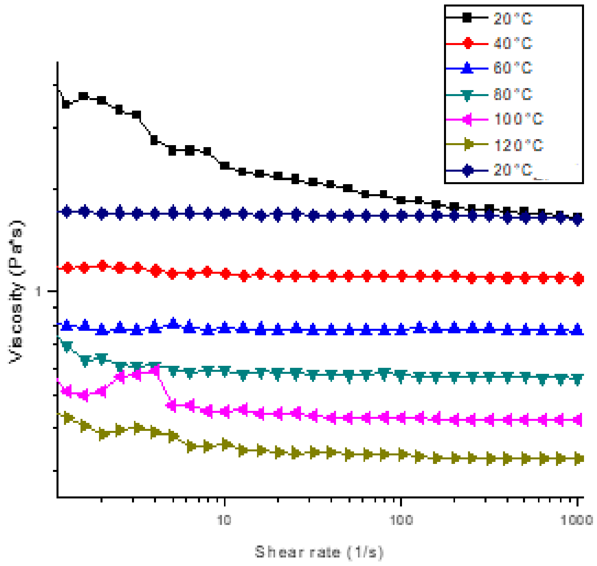

Figure S15. Flow curves of St-128-114 at temperatures from 20 to 120 °C and re-measured at 20 °C after cooling from 120 °C
